# Comparative genomic analysis of metal-tolerant bacteria reveals significant differences in metal adaptation strategies

**DOI:** 10.1128/spectrum.01680-24

**Published:** 2025-04-24

**Authors:** Dai Di Chen, Liu Lian Zhang, Jiu Hua Zhang, Wen Ting Ban, Qingxin Li, Jin Chuan Wu

**Affiliations:** 1 Guangdong Engineering Technology Research Center of Enzyme and Biocatalysis, Institute of Biological and Medical Engineering, Guangdong Academy of Sciences, Guangzhou, China; University of Mississippi, University, Mississippi, USA

**Keywords:** metal-tolerant bacteria, genomic analysis, metal-stress-response, combined use, application

## Abstract

Metal-tolerant bacteria have been commercially used in wastewater treatment, bio-fertilizer, and soil remediation, etc. However, the mechanisms underlying their actions are not yet fully understood. We isolated metal-tolerant bacteria from the rhizosphere soil samples with metal-enriched media containing Cu, Fe, or Mn, sequenced and compared the genomes, and analyzed their metal adaptation strategies at genomic levels to better understand their action mechanisms. Totally, 32 metal-tolerant isolates were identified and classified into 12 genera based on phylogenetic analysis. The determination of maximum tolerance concentration and the effect of metal ions on the isolates indicated that *Serratia marcescens* X1 (CuSO_4_: 1,000 mg/L, FeSO_4_: 1,000 mg/L, and MnSO_4_.4H_2_O: 2,000 mg/L), *Mammaliicoccus sciuri* X26 (FeSO_4_: 600 mg/L and MnSO_4_.4H_2_O: 2,000 mg/L), and *Rummeliibacillus pycnus* X33 (CuSO_4_: 400 mg/L, FeSO_4_: 1,000 mg/L, and MnSO_4_.4H_2_O: 800 mg/L) showed significant differences in metal tolerance to Cu, Fe, and Mn with other isolates. They possess quite different genomic features that enable them to adapt to various metal ions. *S. marcescens* X1 possesses abundant genes required for Cu, Fe, and Mn homeostasis. *M. sciuri* X26 has a number of genes involved in Mn and Zn homeostasis but with no genes responsible for Cu and Ca transport. *R. pycnus* X33 is rich in Fe, Zn, and Mg transport systems but poor in Cu and Mn transport systems. It is thus inferred that the combined use of them would compensate for their differences and enhance their ability in accumulating a wider range of heavy metals for promoting their applications in industry, agriculture, and ecology.

**IMPORTANCE:**

Metal-tolerant bacteria have wide applications in environmental, agricultural, and ecological fields, but their action strategies are not yet fully understood. We isolated 32 metal-tolerant bacteria from the rhizosphere soil samples. Among them, *Serratia marcescens* X1, *Mammaliicoccus sciuri* X26, and *Rummeliibacillus pycnus* X33 showed significant differences in metal tolerance to Cu, Fe, and Mn with other isolates. Comparative genomic analysis revealed that they have abundant and different genomic features to adapt to various metal ions. It is thus inferred that the combined use of them would compensate for their differences and enhance their ability to accumulate heavy metal ions, widening their applications in industry, agriculture, and ecology.

## INTRODUCTION

Metal-tolerant bacteria that exhibit high resistance to metal ions have been found in varied environments and habitats such as water, soil, plants, and humans/animals (1–4). In the recent studies, *Enterobacter* spp., *Pseudomonas* spp., *Leclercia* spp., and *Serratia odorifera* from fresh produce showed good tolerance to copper (Cu) (minimum inhibitory concentration [MIC], 48 mM–60 mM) and zinc (Zn) (MIC, 8 mM–16 mM) (2); *Bacillus* spp., *Rhodococcus equi*, *Serratia marcescens,* and *Paenibacillus chibensis* from water and soil samples collected from coal mine exhibited high maximum tolerable concentration (MTC) of 100 ppm–500 ppm against iron (Fe), of 160 ppm–830 ppm against manganese (Mn), and of 360 ppm–1,400 ppm against plumbum (Pb) (3). Metal-tolerant bacteria possess various metal transport systems to facilitate them as intermediate storage stations for metal ions to shuttle through various environments and habitats. Therefore, metal-tolerant bacteria have been applied in many fields such as treatment of wastewater, transport of metal ions between plants and soil, regulation of soil pH, and remediation of contaminated soil (5–8). With the increasing roles played by metal-tolerant bacteria in human life, recently multi-omics technologies (e.g., genomics, transcriptomics, proteomics, and metabolomics) have been applied for investigating the metal resistance mechanisms of microorganisms (9–12).

Metal ions are required as essential nutrients for the survival of organisms because they play important roles in cellular metabolisms. It is noteworthy that excessive metal ions can have significantly harmful effects on living organisms (13, 14). To survive in environments of varied metal concentrations, microorganisms need diverse strategies to maintain intracellular metal homeostasis. Studies on mechanisms of metal homeostasis in microorganisms suggest that metal transport systems and metal-binding proteins are key players in response to metal stress (15, 16). In bacteria, metal transport systems are conserved but diverse, such as ATP-binding cassette (ABC) transporters (e.g., *Actinobacillus* ferric uptake system AfuABC required for Fe influx [17], *Salmonella* iron transporter SitABCD involved in the acquisition of Fe and Mn [18], Mn transporter MntABC responsible for Mn uptake [19], and Zn uptake system ZnuABC necessary for Zn import [15]), two-component system (e.g., CusSR and PcoSR regulating Cu influx [20], PhoPQ mediating magnesium [Mg] import [21], and potassium (K)-dependent protein KdpDE controlling K uptake [22]), P-type ATPases (e.g., CopA contributing to the Cu efflux [20]), symporter (e.g., glutamate:Na^+^ symporter GltS, solute:Na^+^ symporter SSS, and proline:Na^+^ symporter PutP performing sodium [Na] influx [23]), and antiporter (e.g., Na^+^:H^+^ antiporter NhaABC and multicomponent Na^+^:H^+^ antiporter MnhA-G carrying out Na efflux [24], and Ca^2+^:H^+^ antiporter ChaA undertaking calcium (Ca) export [25]).

In order to obtain metal-tolerant bacteria, we screened the isolates from rhizosphere soil samples with metal-enriched medium containing Cu, Fe, or Mn and performed phylogenetic analysis based on the 16S ribosomal RNA (rRNA) sequences of the isolates. Moreover, we sequenced and compared their genomes, analyzed their metal adaptation strategies at the genomic level to provide insight into their differences in action mechanisms for guiding the development of their new applications.

## RESULTS AND DISCUSSIONS

### Isolation and molecular identification of metal-tolerant bacteria

Nearly 100 isolates from rhizosphere soil samples were screened using the metal-enriched medium containing 200 mg/L each of CuSO_4_, FeSO_4_, or MnSO_4_.4H_2_O. Totally, 32 isolates were identified with 16S rRNA gene sequences revealing their phylogenetic affiliation with 12 genera, including *Pseudomonas*, 8; *Cupriavidus*, 5; *Enterobacter*, 4; *Klebsiella*, 4; *Bacillus*, 3; *Chryseobacterium*, 2; *Atlantibacter*, 1; *Lelliottia*, 1; *Exiguobacterium*, 1; *Rummeliibacillus*, 1; *Serratia*, 1; and *Mammaliicoccus,* 1. The Fe-, Mn-, and/or Pb-tolerant bacteria, which were isolated from coal mine samples, belong to the genera of *Bacillus*, *Serratia*, *Rhodococcus,* and *Paenibacillus* (3). Cidre et al. isolated the Cu- and Zn-resistant bacteria from fresh produce and identified them to six genera: *Enterobacter*, *Leclercia*, *Pseudomonas*, *Serratia*, *Bacillus,* and *Paenibacillus* (2). These results suggest that the species of *Enterobacter*, *Pseudomonas*, *Serratia,* and *Bacillus* might act as the key players in the transformation of metal (Fe, Cu, or/and Mn) tolerance in soil and plants.

**Fig 1:**
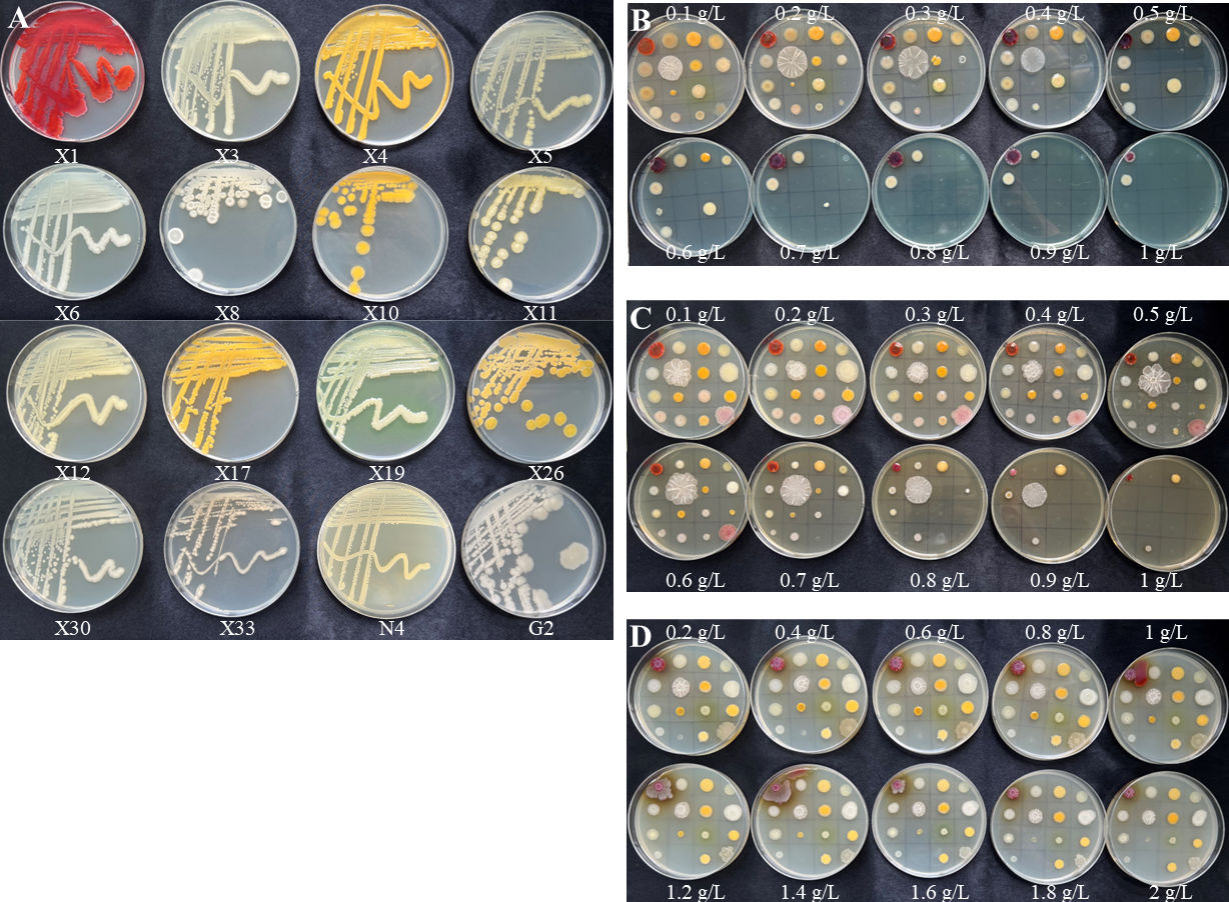
MTC of 16 metal-tolerant bacterial isolates. Sixteen isolates were grown on Luria-Bertani (LB) medium (**A**). Sixteen isolates were grown on LB medium supplemented with various concentrations of CuSO_4_ (**B**), FeSO_4_ (**C**), and MnSO_4_.4H_2_O (**D**). Photographs were taken 3 days after inoculation at 30°C.

**Fig 2:**
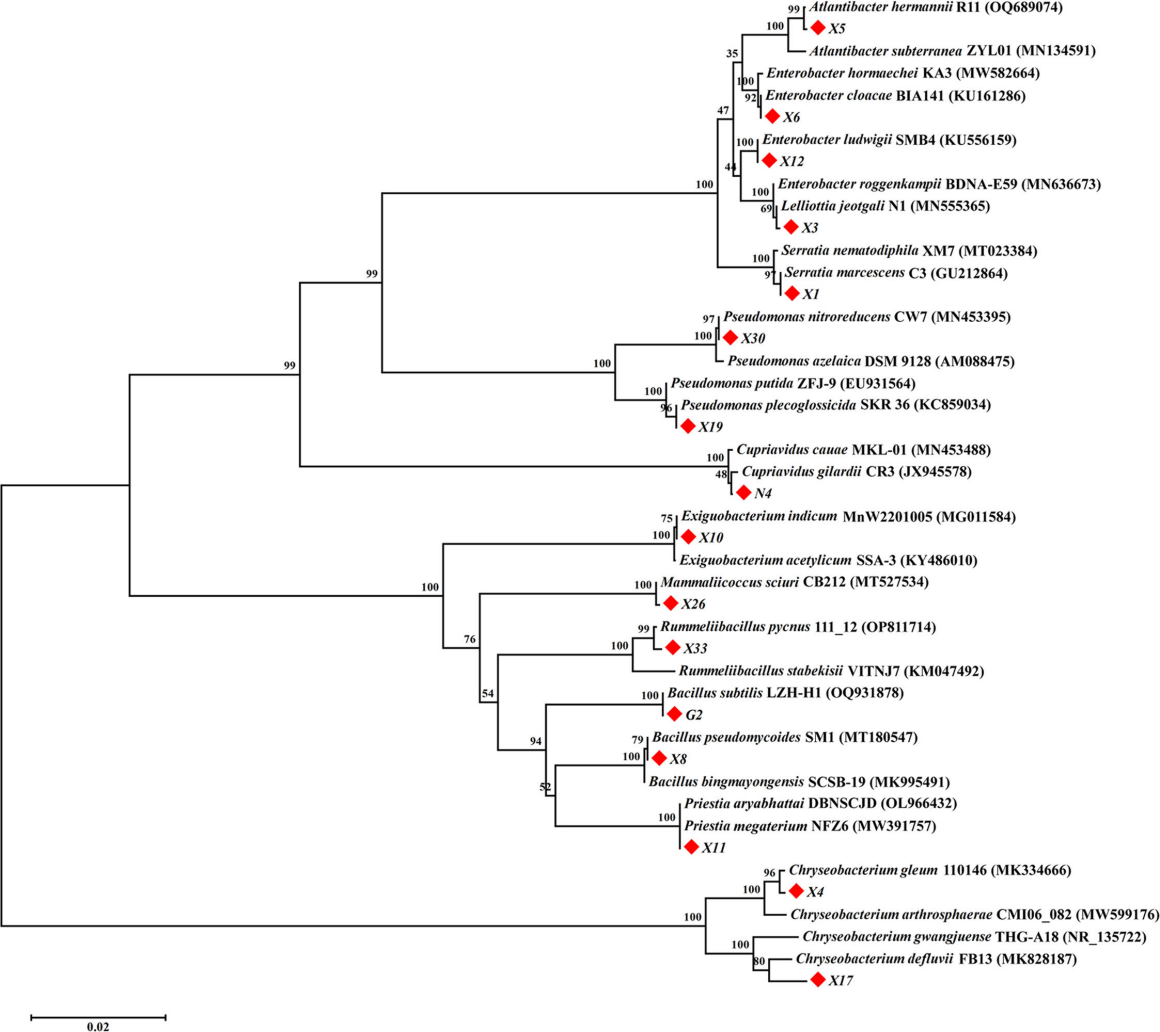
Phylogenetic relationships of 16 metal-tolerant bacterial isolates and the related species. Neighbor-joining phylogenetic tree based on 16S rRNA gene sequences showing the phylogenetic positions of 16 metal-tolerant bacterial isolates (indicated with solid red diamond). The bootstrap values are shown at branch nodes. Bar, 0.02 nucleotide position.

Among the 32 isolates, 16 of them exhibited distinct colony morphologies and the ones that were at least tolerant to two metals were selected for further investigation (Fig. 1). The phylogenetic relationships of the 16 isolates and the related species were analyzed using a neighbor-joining phylogenetic tree with their 16S rRNA gene sequences (Fig. 2; Table 1). A 16S rRNA gene identity >98% is a traditional criterion for delineating microbial species, suggesting that two strains are of the same species (26). The isolates X1, X3, and X5 showed 16S rRNA gene sequence similarities of 99.72%, 99.65%, and 99.93% with *Serratia marcescens* C3, *Lelliottia jeotgali* N1, and *Atlantibacter hermannii* R11, respectively, so the isolates X1, X3, and X5 were identified as *S. marcescens*, *L. jeotgali,* and *A. hermannii*. The isolates X4 and X17 were homologous to *Chryseobacterium* spp. (>98.43% similarities), so they were classified as *C. gleum* and *C. defluvii*, respectively. The isolates X6 and X12 clustered together with *Enterobacter* spp. by >99.72% of 16S rRNA gene identities, so they were identified as *E. cloacae* and *E. ludwigii*, respectively. The isolates X8, X11, and G2 were most matching with *Bacillus* spp. with 16S rRNA gene sequence similarities of >99.79%, so they were designated as *B. pseudomycoides* X8, *Priestia megaterium* X11, and *B. subtilis* G2, respectively. The 16S rRNA gene sequences of isolates X10 and N4 were 99.79% and 99.38% similar to those of *Exiguobacterium indicum* MnW2201005 and *Cupriavidus gilardii* CR3, so they were classified as *E. indicum* and *C. gilardii*, respectively. The 16S rRNA gene sequences of isolates X19 and X30 correlated well with those of *Pseudomonas* spp. (>99.72%), so they were identified as *P. plecoglossicida* and *P. nitroreducens*, respectively. The isolates X26 and X33 were closest to *Mammaliicoccus sciuri* CB212 and *Rummeliibacillus pycnus* 111_12 with great bootstrap supports (100% and 99%) and 16S rRNA gene sequence similarities (99.72% and 99.08%, respectively). Therefore, they were designated as *M. sciuri* X26 and *R. pycnus* X33, respectively.

**TABLE 1:** 16S rRNA gene identities of metal-tolerant bacterial isolates with related species

Isolates	GenBank no.	Related species	16S rRNA gene identities
X1	OR342587	*Serratia marcescens* C3	99.72%
X3	OR342588	*Lelliottia jeotgali* N1	99.65%
X4	OR342589	*Chryseobacterium gleum* 110146	99.24%
X5	OR342590	*Atlantibacter hermannii* R11	99.93%
X6	OR342591	*Enterobacter cloacae* BIA141	99.72%
X8	OR342592	*Bacillus pseudomycoides* SM1	99.79%
X10	OR342593	*Exiguobacterium indicum* MnW2201005	99.79%
X11	OR342594	*Priestia megaterium* NFZ6	99.86%
X12	OR342595	*Enterobacter ludwigii* SMB4	100%
X17	OR342596	*Chryseobacterium defluvii* FB13	98.43%
X19	OR342597	*Pseudomonas plecoglossicida* SKR36	99.72%
X26	OR342598	*Mammaliicoccus sciuri* CB212	99.72%
X30	OR342599	*Pseudomonas nitroreducens* CW7	99.79%
X33	OR342600	*Rummeliibacillus pycnus* 111_12	99.08%
N4	OR342601	*Cupriavidus gilardii* CR3	99.38%
G2	OR342602	*Bacillus subtilis* LZH-H1	100%

### Maximum tolerance concentration (MTC) of metal-tolerant bacteria

The MTC of the 16 isolates for Cu, Fe, and Mn was determined using the spot plate method, and the results were shown in Fig. 1B through D. For Cu, the highest MTC (1,000 mg/L) was exhibited by *S. marcescens* X1 and *E. cloacae* X6, followed by *L. jeotgali* X3 and *A. hermannii* X5 (900 mg/L). Besides, all isolates could tolerate a minimum concentration of 200 mg/L of CuSO_4_, except for *M. sciuri* X26. In the case of Fe, *S. marcescens* X1, *C. gleum* X4, and *R. pycnus* X33 displayed the highest MTC of 1,000 mg/L, accompanied by *E. cloacae* X6 and *B. pseudomycoides* X8 (MTC of 900 mg/L). Moreover, all isolates could tolerate a minimum concentration of 600 mg/L of FeSO_4_. All isolates could grow at a concentration of 2,000 mg/L of MnSO_4_.4H_2_O, except for *R. pycnus* X33, which showed an MTC of 400 mg/L against Mn. Overall, *S. marcescens* X1 exhibited the highest MTC for Cu, Fe, and Mn, *M. sciuri* X26 showed the highest MTC for Mn and the lowest MTC for Cu, and *R. pycnus* X33 displayed the highest MTC for Fe and the lowest MTC for Mn. As the above three isolates showed significant differences with others, they were selected for further investigation.

*Enterobacterales* have been reported existing in multiple niches including soil, water, plants, animals, and humans. Many species of this order have exhibited high resistance to multiple metals and antibiotics (2–4). It is worth noting that the isolates belonging to the *Enterobacterales* order (*S. marcescens* X1, *L. jeotgali* X3, *A. hermannii* X5, and *E. cloacae* X6) exhibited superior tolerance to multiple metals. The isolate *S. marcescens* X1 showed higher tolerance to metals than other reported *S. marcescens* strains such as *S. marcescens* KH-CC (MTC of 500 ppm against Fe and 830 ppm against Mn) (3), *S. marcescens* CL11 and *S. marcescens* CL35 tolerated 1,200 mg/L of Mn (27). Shylla et al. reported that *B. pseudomycoides* KH-12A showed 400 ppm tolerance to Fe and 730 ppm to Mn, obviously lower than those of *B. pseudomycoides* X8 (3). The MTC values of 90 ppm–1,000 ppm against Fe and MTC of 120 ppm–800 ppm against Mn have been reported in other *Bacillus* species (3, 28), while the isolates *P. megaterium* X11 tolerated 800 mg/L of Fe and 2,000 mg/L of Mn, and *B. subtilis* G2 resisted 600 mg/L of Fe and 2,000 mg/L of Mn.

### Effect of metals on bacterial growth

**Fig 3:**
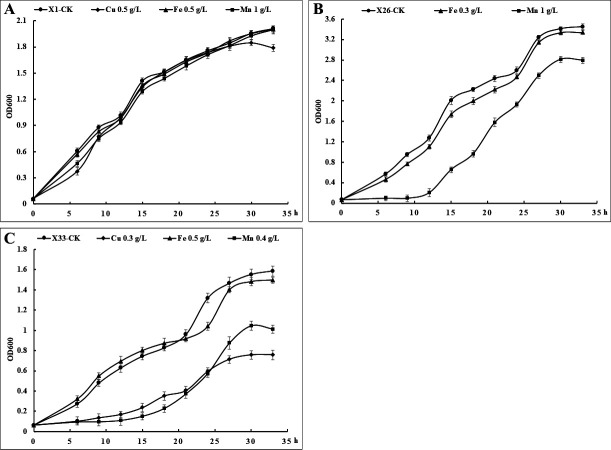
Effect of metals on bacterial growth. *S. marcescens* X1 (**A**), *M. sciuri* X26 (**B**), and *R. pycnus* X33 (**C**) in the presence of Cu, Fe, and Mn.

The growth kinetics of the isolates X1, X26, and X33 cultured on LB broth with middle concentrations of metals were monitored to investigate the effects of metals (Cu, Fe, and Mn) on bacterial growth. A nonsignificant difference was observed in the growth curves of isolate X1 at non-metals, 500 mg/L of CuSO_4_, 500 mg/L of FeSO_4_, and 1,000 mg/L of MnSO_4_.4H_2_O, reaching an OD_600_ of approximately 1.9 after 30 h (Fig. 3A), indicating that the middle concentrations of Cu, Fe, and Mn for isolate X1 did not obviously affect the growth of this isolate. As the isolate X26 could not resist Cu, which was evidenced from the result of the MTC test, the effect of Cu was not carried out here. Besides, the isolate X26 reached OD_600_ values of 3.41, 3.34, and 2.81 at non-metals, 300 mg/L of FeSO_4_, and 1,000 mg/L of MnSO_4_.4H_2_O after 30 h, respectively (Fig. 3B), suggesting that the middle concentration of Fe for isolate X26 slightly inhibited its growth, while the middle concentration of Mn for isolate X26 significantly inhibited its growth. The isolate X33 grew in LB broth containing 500 mg/L of FeSO_4_ after 30 h giving an OD_600_ of 1.48, which is close to the case of the absence of metals (OD_600_ = 1.55) (Fig. 3C). In contrast, the isolate got lower growth rates with OD_600_ values of 0.76 and 1.04 at 300 mg/L of CuSO_4_ and 1,000 mg/L of MnSO_4_.4H_2_O after 30 h, respectively (Fig. 3C). These results suggest that Cu has the strongest inhibitory effect on the growth of isolate X33, followed by Mn. Overall, the isolate X1 could resist various metals at high concentrations, and its metal tolerance ability is the strongest, followed by isolate X33, then isolate X26. In addition, Cu is most toxic to the isolates, followed by Mn, then Fe.

### Genomic characteristics of the three metal-tolerant bacterial isolates

**Fig 4:**
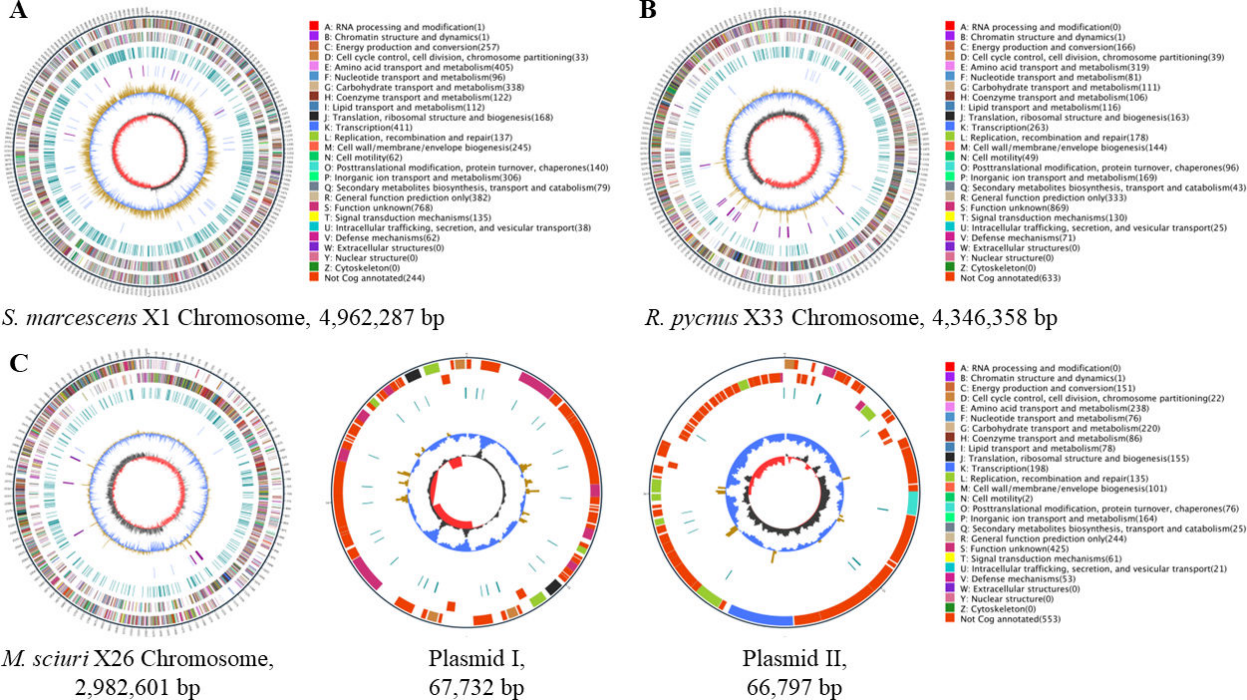
Circular maps of *S. marcescens* X1 (**A**), *R. pycnus* X33 (**B**), and *M. sciuri* X26 (**C**) genomes. The numbers 1 to 7 are the circles of circular maps from inner to outer: (1) GC skew. Dark gray represents areas with G content greater than C, while red represents areas with C content greater than G. (2) GC content. The value is plotted as the deviation from the average GC content of the entire sequence. (3) tRNA (blue) and rRNA (purple). (4) Repetitive sequences. (5, 6) CDS colored according to COG functional categories, 5 is negative strand, 6 is positive strand. (7) Marker of genome size, with each scale measuring 5 kb.

Genomic features of the three metal-tolerant bacterial isolates are shown in Fig. 4 and Table 2. The complete genome of *S. marcescens* X1 is assembled in a single circular chromosome of 4,962,287 bp (G+C content of 59.82%), which is smaller than that of other *S. marcescens* strains such as N2 (5.14 Mbp), RSC-14 (5.13 Mbp), and WW4 (5.24 Mbp) (10, 29). A total of 4,542 genes were predicted from the genome of *S. marcescens* X1, including 92 transfer RNA (tRNA) genes and 22 rRNA genes. The draft genome of *M. sciuri* X26 (3,117,130 bp with a G+C content of 32.56%) assembly contains a complete circular chromosome (2,982,601 bp) and two plasmids (plasmid I: 67,732 bp and plasmid II: 66,797 bp). There are 3,085 genes in the genome, which include 58 tRNA genes and 19 rRNA genes. Compared with *M. sciuri* X26, *M. sciuri* IMDO-S72 has a smaller size of genome (2,898,343 bp) containing a chromosome and four plasmids and fewer genes (2,923) (30). The final genome of *R. pycnus* X33 is a complete circular chromosome with a size of 4,346,358 bp and a GC content of 34.97%, which is bigger than that of *R. pycnus* DSM 15030 (3,851,193 bp) (NJAS01000000). Totally, 4,105 predicted genes were found in the genome of *R. pycnus* X33, of which 122 are tRNA genes and 39 are rRNA genes. In addition, 4,532, 3,046, and 3,971 coding sequences (CDSs) were functionally annotated with Cluster of Orthologous Groups (COG), Gene Ontology (GO), Swiss-Prot, Non-Redundant Protein Database (NR), and Kyoto Encyclopedia of Genes and Genomes (KEGG) database from the genome of *S. marcescens* X1, *M. sciuri* X26, and *R. pycnus* X33, respectively (Table S1, DOI 10.17605/OSF.IO/G6728; https://osf.io/g6728/?view_only=80422cbf18ab4173885728f83db99ed5). Functional categories of genes based on the COG database are shown in Fig. 4 and Fig. S1.

**TABLE 2:** Comparative genome statistics of three metal-tolerant bacterial isolates and other strains of homogeneity

Strains/Features	*S. marcescens* X1	*S. marcescens* N2	*M. sciuri* X26	*M. sciuri* IMDO-S72	*R. pycnus* X33	*R. pycnus* DSM 15030
Genome size (bp)	4,962,287	5,140,264	3,117,130	2,898,343	4,346,358	3,851,193
Chromosome	1	1	1	1	1	1
Plasmid	0	0	2	4	0	0
GC content (%)	59.82	59.1	32.56	32.6	34.97	34.5
Genes	4,542	5,501	3,085	2,923	4,105	3,784
CDSs	4,532	5,393	3,046	2,842	3,971	3,625
tRNA	92	86	58	58	122	116
rRNA (5S/16S/23S)	8/7/7	2/2/2	7/6/6	7/6/6	13/13/13	13/12/12
GenBank accession number	CP132290 (this study)	SPSG02000000	JAUZEF000000000 (this study)	CAJVGN010000000	CP132291 (this study)	NJAS01000000

### Metal adaptation strategies of the three metal-tolerant bacterial isolates

**Fig 5:**
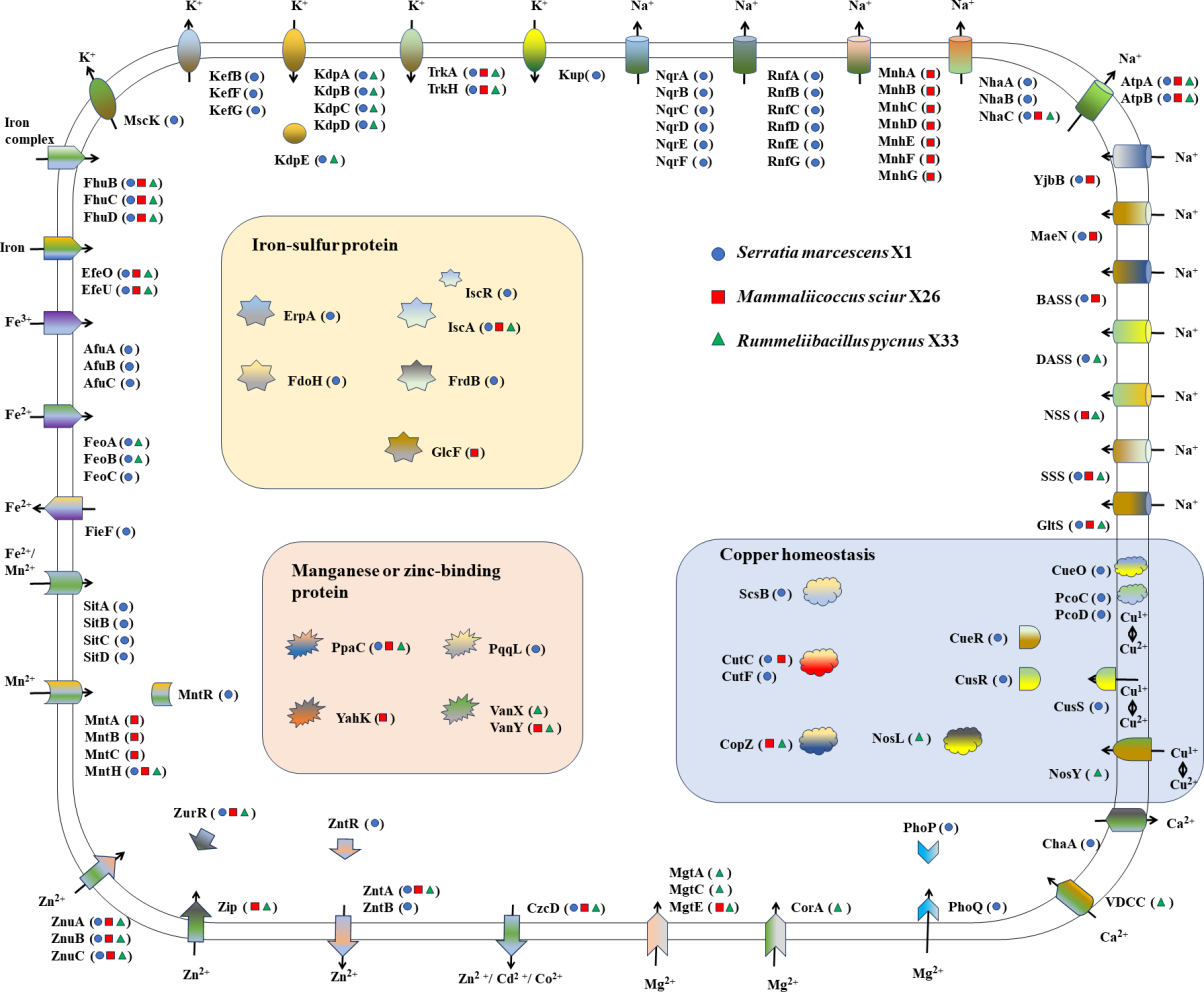
Schematic of the proteins identified as playing a putative role in the adaptation of three metal-tolerant bacterial isolates to metal environments. Strains: *S. marcescens* X1 (blue solid circle), *M. sciuri* X26 (red solid square), and *R. pycnus* X33 (green solid triangle). The putative function of genes is shown in Table S2.

Metal ions are essential micronutrients for biological systems, forming active centers of various metalloenzymes. However, excess amounts of metals are cytotoxic. Therefore, elucidation of the mechanisms of metal responses in bacteria would help develop sophisticated cultivation strategies for new applications. To investigate the metal adaptation strategies of metal-tolerant bacteria at a genomic level, the genes responsible for metal tolerance pathways in the genomes of the three metal-tolerant bacterial isolates were identified and compared (Fig. 5 and Table S2). It is seen that the putative metal stress-response genes are different in both types and amounts, which likely results in their different metal adaptation strategies.

For copper homeostasis in *S. marcescens* X1, the homologs of a multi-copper oxidase, CueO, and two periplasmic chelators, PcoC and PcoD, probably perform copper detoxification in the periplasm by converting periplasmic Cu(I) to Cu(II) (31, 32). The Cu(I) in periplasm fluxes across the inner membrane under the control of the homologs of three sensory systems, i.e., the MerR-family transcriptional regulator (CueR) and the CusS/CusR two-component system (20). The Cu(I) in the cytoplasm may be scavenged by the homologs of a high-affinity cuprochaperone (CopZ) and transported by two copper homeostasis proteins (CutC and CutF) (20, 33). *S. marcescens* X1 shows high copper resistance most likely due to the richness of genes involved in copper homeostasis in its genomes. *M. sciuri* X26 possesses CopZ and CutC but lacks the copper sensory systems, likely resulting in conditional lethality in the presence of exogenous copper. The copper tolerance of *R. pycnus* X33 (MTC of 400 mg/L) was lower than *S. marcescens* X1 (MTC of 1,000 mg/L), likely owing to the poorer copper response systems, i.e., two copper chaperones (CopZ and NosL) and one Cu-processing system permease protein (NosY). It is noteworthy that the copper resistance genes (i.e., *cusSR* and *pcoCD*) were not found in *M. sciuri* X26 and *R. pycnus* X33, but were only found in *S. marcescens* X1. The *cus* and *pco* gene clusters and accessory genes situated between transposable elements are composed of a copper homeostasis and silver resistance island (CHASRI), which was found on chromosomes in *Enterobacteriaceae* (such as *Escherichia coli*, *E. cloacae*, *Klebsiella pneumoniae*, *Cronobacter turicensis*, *Cronobacter sakazakii,* and *S. marescens* strains which are facultative anaerobic human or animal pathogens) (2, 34). Staehlin et al. proposed that the CHASRI had originated from a relative of *E. cloacae* and subsequently experienced dispersal among *Enterobacteriaceae* by horizontal gene transfer (HGT). Moreover, the resistance function of CHASRI was increased during shifts between aerobic and anaerobic environments; thus, CHASRI could be maintained within facultative anaerobes of human or animal pathogens (34).

In the *S. marcescens* X1 genome, a number of genes encoding the homologs of proteins responsible for Fe uptake pathways were discovered, including the ferric hydroxamate uptake (Fhu) system (FhuB, C, and D) necessary for the transport of ferrichrome, and other Fe^3+^ compounds from the periplasm (35), elemental Fe(II/III) (Efe) complex (EfeO and EfeU) required for the acquisition and utilization of iron (36), the AfuABC system responsible for the uptake of Fe(III) (17), the ferrous iron transport (Feo) system (FeoA, B, and C) involved in the transport of Fe(II) (37), and the *Salmonella* iron transporter (Sit) (SitA, B, C, and D) which plays an important role in Fe acquisition under iron-deficient conditions (38). Many iron-sulfur proteins, i.e., iron-sulfur cluster assembly protein/iron-sulfur cluster assembly regulator, A-type iron-sulfur carrier protein (ErpA), fumarate reductase iron-sulfur subunit (FrdB), and formate dehydrogenase iron-sulfur subunit (FdoH), have been reported to play important roles in Fe homeostasis and many cellular processes (39–42). Therefore, the determination of MTC and the effect of iron on *S. marcescens* X1 show that this isolate exhibited high tolerance to iron (MTC of 1,000 mg/L), and its growth was not obviously affected by middle concentration of iron (500 mg/L of FeSO_4_). *R. pycnus* X33 also exhibited an MTC of 1,000 mg/L against Fe, but its growth obviously decreased under 500 mg/L FeSO_4_, which might be ascribed to the absence of some Fe uptake systems (i.e., AfuABC system and SitABCD transporters) and iron-sulfur proteins (ErpA, FrdB, and FdoH) in *R. pycnus* X33 compared with *S. marcescens* X1. *M. sciuri* X26 appears to absorb iron only via two Fe uptake systems, FhuBCD system and EfeUO complex, resulting in lower copper tolerance than the other two isolates.

*S. marcescens* X1 exhibited high tolerance to Mn (MTC of 2,000 mg/L), possibly due to the two types of Mn import systems present in this isolate. The ﬁrst system is an H^+^-dependent Mn^2+^ transporter, MntH, which is a high-affinity manganese acquisition system regulated by MntR (16, 18). The second system is an ABC transporter, SitABCD, which transports Mn also via the mediation of MntR (18). *M. sciuri* X26 showed high tolerance to Mn (MTC of 2,000 mg/L), possibly resulting from two manganese transporters, i.e., homologs of MntH and ABC transporter MntABC (19). However, the absence of MntR might reduce the Mn tolerance of this isolate, so its growth decreased under 1,000 mg/L MnSO_4_.4H_2_O. The Mn tolerance of *R. pycnus* X33 was lowest among the three isolates, which might be attributed to the only one manganese transporter (MntH homolog) in this isolate. The MntH homologs were found in all three isolates, X1, X26, and X33. The study of Cellier revealed that aerobic bacteria (such as *Firmicutes*, *Actinobacteria*, *Deinococcus,* and *Enterobacteria*) MntH dispersed via HGT within bacterial solute carriers 11 (a family of metal-ion transporters playing an important role in nutrition and host-microbe interactions), resulting in the enhancement of bacterial stress resistance function by aerobic metabolism (43).

In the genomes of the three isolates, a number of genes involved in the transportation and homeostasis of other metals (i.e., Zn, Mg, Ca, Na, and K) were also found. For the transportation of Zn, the Znu and Zip (Zrt/Irt-like protein) systems are believed to be responsible for the influx of Zn into the cells, while the Znt (Zn transporter) and Czc (cobalt, zinc, and cadmium) systems are thought to be involved in the efflux of Zn out of the cells (15, 16, 44). For Zn uptake, the homologs of *znuABC* and their regulatory gene *zurR* were found in the genomes of the three isolates, while the homolog of *zip* is only present in the genomes of *M. sciuri* X26 and *R. pycnus* X33. For the Zn efflux, *S. marcescens* X1 possesses the homologs of ZntABR and CzcD, while *M. sciuri* X26 and *R. pycnus* X33 only have ZntB and CzcD homologs. Moreover, some genes, *vanX* (encoding zinc-dependent D-Ala-D-Ala dipeptidase), *vanY* (encoding zinc-containing D-Ala-D-Ala carboxypeptidase), and *yahK* (encoding zinc-type alcohol dehydrogenase-like protein), involved in the Zn homeostasis and many physiological processes (45, 46), were found in the genomes of *M. sciuri* X26 and/or *R. pycnus* X33 but not in the genome of *S. marcescens* X1. These findings suggest that *M. sciuri* X26 and *R. pycnus* X33 may have a superior ability for resisting Zn compared to *S. marcescens* X1. The main Mg transport systems of the three isolates show differences. The Mg uptake of *S. marcescens* X1 is likely mediated by the homologs of PhoP (the response regulator/transcriptional activator)/PhoQ (the sensor/receptor histidine kinase) two-component system (21). In *M. sciuri* X26, the Mg import into cells may be performed by transporter MgtE, a widely distributed Mg^2+^ channel in microorganisms (47). The four Mg transporters, CorA, MgtA, MgtB, and MgtE, reported to be highly selective for Mg uptake (48), probably contribute to the Mg transport in *R. pycnus* X33. In the case of Ca transport, one homolog of VDCC (voltage-dependent Ca^2+^-channel) involved in the Ca^2+^ influx (49) was only found in *R. pycnus* X33, and one homolog of ChaA responsible for the Ca^2+^ efflux (25) is only present in *S. marcescens* X1.

It is well known that the intracellular concentrations of K and Na are very important to the physiological processes and salt stress response in microorganisms. In microorganisms, the K^+^ influx is mainly performed by four systems, the Trk/Ktr (K-transport), Kdp, and Kup (K^+^ uptake) systems, while the K^+^ efflux is mainly carried out by the glutathione-gated potassium (K) efflux (Kef) system and mechanosensitive channels (Msc) (23). Among these K^+^ uptake systems, Trk and Kup are constitutive systems, while Kdp is an inducible system, exhibiting high affinity for K^+^ (50). In *S. marcescens* X1, three K^+^ influx systems, KdpABCDE, TrkAH, and Kup, were found, and in *R. pycnus* X33, two K^+^ uptake systems, KdpABCDE and TrkAH, were discovered, while in *M. sciuri* X26, only TrkAH was observed. It is noteworthy that *S. marcescens* X1 possesses the homologs of KefBFG and MscK for K^+^ efflux, but no homologs of Kef or Msc were found in *M. sciuri* X26 and *R. pycnus* X33. It is likely that *M. sciuri* X26 and *R. pycnus* X33, as well as *S. marcescens* X1, do not have a strong ability for K^+^ uptake, so they require reducing K^+^ efflux to maintain intracellular K homeostasis. In microorganisms, the uptake of Na is performed by the important secondary active transporters (Na^+^-symporters) (23). In the genomes of the three isolates, some genes homologous to Na^+^-symporters, i.e., glutamate:Na^+^ symporter (*gltS*), solute:Na^+^ symporter (*sss*), neurotransmitter:Na^+^ symporter (*nss*), divalent anion:Na^+^ symporter (*dass*), bile acid:Na^+^ symporter (*bass*), malate:Na^+^ symporter (*maeN*), and phosphate:Na^+^ symporter (*yjbB*) were found. Except for the homolog of NSS, the other six Na^+^-symporters were found in *S. marcescens* X1, while only the homolog of *dass* is not present in the genome of *M. sciuri* X26. Correspondingly, in *S. marcescens* X1 and *M. sciuri* X26, there are many systems performing Na^+^ efflux to maintain intracellular Na homeostasis, including Na^+^-translocating NADH:ubiquinone oxidoreductase complex (NqrA-F), Na^+^-translocating NAD^+^:ferredoxin oxidoreductase complex (RnfA-E and RnfG), F-type H^+^:Na^+^-translocating ATPase complex (AtpAB), NhaABC, and/or MnhA-G (24, 51–53). Different from *S. marcescens* X1 and *M. sciuri* X26, *R. pycnus* X33 only has four Na^+^-symporters, i.e., GltS, SSS, NSS, and DASS, and the Na^+^ efflux is only carried by AtpAB and NhaC.

In conclusion, 32 metal-tolerant isolates screened from the rhizosphere soil samples with metal-enriched media containing Cu, Fe, or Mn were identified and classified into 12 genera. The determination of MTC and the effect of metals on the metal-tolerant isolates indicated that among them, *S. marcescens* X1, *M. sciuri* X26, and *R. pycnus* X33 showed significant differences in metal tolerance to Cu, Fe, and Mn with other isolates. *S. marcescens* X1 could resist high concentrations of Cu, Fe, and Mn, *M. sciuri* X26 could resist high content of Mn but not Cu, and *R. pycnus* X33 could tolerate high levels of Fe but not Mn. To investigate their metal adaptation strategies at a genomic level, the genomes of the three isolates were sequenced and compared. It was found that the genomic features which have been believed to play an important role in adapting to metal environments are significantly different for the three isolates. *S. marcescens* X1 possesses a number of genes encoding transporters, channels, and metal-binding proteins to adapt to the environments containing high concentrations of Cu, Fe, and Mn. *M. sciuri* X26 has a number of genes involved in Mn and Zn homeostasis but no genes responsible for Cu and Ca transport. *R. pycnus* X33 is rich in Fe, Zn, and Mg transport systems but poor in Cu and Mn transport systems. Based on these findings, *S. marcescens* X1 is expected to serve as an eco-friendly biosorbent or biological material for effectively removing Cu, Fe, and Mn from wastewater and polluted soil. *M. sciuri* X26 is predicted to be used for treating the environment containing high amounts of Mn and Zn, and *R. pycnus* X33 is desired for accumulating Fe, Zn, and Mg from the environment. The combined use of them might serve as efficient plant growth regulators for the transport of metals between plants and soil as well as for the regulation of soil pH. Additionally, *S. marcescens* X1 might play a role in quick restoration of salt-contaminated soils considering its abundant K and Na transport systems, which were found in halophilic and halotolerant microbes. The aim of this study is to provide a preliminary insight into the differences of metal-tolerant bacteria in their action mechanisms to guide their combined use for new applications. In our subsequent work, we will further investigate the metal tolerance and phylogeny of microbial communities in contaminated soils so as to provide a more holistic understanding of the different strategies of the microbes in coping with metal stresses.

## MATERIALS AND METHODS

### Source and isolation of metal-tolerant bacteria

The soil samples were collected from the rhizosphere of banana and sugarcane grown at Guangzhou, Guangdong province, southern China (113.84°E, 23.31°N), and stored aseptically in a refrigerator at 4°С until processed for isolation. The soil samples (5 g) were diluted to a concentration of 10^−3^ and 10^−4^ (wt/vol) with autoclaved distilled water. The dilutes were then spread (100 µL per plate) onto modified Luria-Bertani (LB) agar plates (HKM, Guangdong, Chain) supplemented with 200 mg/L each of CuSO_4_, FeSO_4_, or MnSO_4_.4H_2_O (Macklin, Shanghai, Chain), and the pH was adjusted to 7.2 ± 0.3. The isolates were selected based on varying colony morphology after 2 days of incubation at 30°C, then purified, cultivated, and maintained on the same medium. Pure cultures were preserved as 20% (wt/vol) glycerol at −80°С.

### Phylogenetic analysis of metal-tolerant bacterial isolates

The identification of metal-tolerant bacterial isolates was performed by sequencing the 16S rRNA genes. The 16S rRNA gene was amplified with forward 27F (5′-AGAGTTTGATCCTGGCTCAG-3′) and reverse 1492R (5′-TACCTTGTTACGACTT-3′) primers, and sequenced on an Illumina platform (Illumina NovaSeq 6000) at Tsingke Biotech (Beijing, China). The partial sequences of the 16S rRNA gene sequences of the metal-tolerant bacterial isolates were deposited in GenBank under the accession numbers OR342587–OR342602. For phylogenetic analysis, 16S rRNA sequences of type strains with validly published prokaryotic strains affiliated with the isolates were retrieved from the GenBank database. The 16S rRNA gene sequences were aligned to produce a phylogenetic tree constructed with 1,000 bootstrap replications via the neighbor-joining method using MEGA 4.0 software (54).

### Determination of MTC for metals

The MTC of the isolates was determined by using the spot plate method with LB agar plates supplemented with CuSO_4_ concentrations ranging from 100 to 1,000 mg/L, FeSO_4_ concentrations ranging from 100 to 1,000 mg/L, and MnSO_4_.4H_2_O concentrations ranging from 100 to 2,000 mg/L (2, 3). The culture of 3 µL (corresponding to 0.5 OD at 600 nm) from each isolate grown overnight was spotted onto the plates and observed for visible growth after 3 days of incubation at 30°C. The MTC was defined as the lowest concentration of metal without visible growth of the test strain.

### Investigation of the effect of metals on bacterial growth

Isolate X1 that exhibited the highest MTC for three metals (Cu, Mn, and Fe), isolate X26 that showed the highest MTC for Mn and the lowest MTC for Cu, and isolate X33 that displayed the highest MTC for Fe and the lowest MTC for Mn were investigated for the effect of metals on their growth. The culture of 100 µL (corresponding to 0.5 OD at 600 nm) from the three strains grown overnight was transferred onto LB broth (100 mL) supplemented with middle concentrations of metals that the isolates could tolerate, i.e., isolate X1: 500 mg/L of CuSO_4_, 500 mg/L of FeSO_4_, and 1,000 mg/L of MnSO_4_.4H_2_O; isolate X26: 300 mg/L of FeSO_4_ and 1,000 mg/L of MnSO_4_.4H_2_O; isolate X33: 300 mg/L of CuSO_4_, 500 mg/L of FeSO_4_, and 1,000 mg/L of MnSO_4_.4H_2_O. The media with bacteria but no metal were used as controls. The growth curves of the strains cultured in liquid medium with varied concentrations of metals at 30°C for 2 days, with shaking at 150 rpm, were produced by determining at OD_600_ using a SpectraMax iD3 microplate reader (Molecular Devices, CA, USA). All experiments were performed three times.

### Genome sequencing, assembly, and prediction

Genomic DNAs were extracted from the isolates X1, X26, and X33 using the GenElute bacterial genomic DNA extraction kit (Sigma-Aldrich, St. Louis, MO), and sequenced on a Nanopore platform (PromethION48) at Biomarker Technologies (Beijing, China). The filtered reads that were removed reads with mean_qscore_template of <7 and length of <2,000 bp from raw reads were assembled by Canu v.1.5 software (55). Racon v.3.4.3 software (56) was used to check the quality of the assembly, and then Circlator v.1.5.5 software was used to cyclize the assembly genome (57). The prediction of coding genes and repetitive sequences was performed by Prodigal v.2.6.3 (58) and RepeatMasker v.4.0.5 (59), respectively. tRNA and rRNA genes were predicted using tRNAscan-SE v.2.0 (60) and Infernal v.1.1.3 (61), respectively. PromPredict v.1 and antiSMASH v.5.0.0 were used for prediction of promoter (62) and secondary metabolic gene clusters (63), respectively.

### Genome annotation and comparison

For functional annotation, the protein sequences of the predicted CDSs were blasted against the databases, including COG, GO, Swiss-Prot, KEGG, and NR. The subsystem analysis was performed by annotation using the subsystems technology server Rapid Annotation using Subsystem Technology (RAST) (64). To investigate the differences in metal adaptation strategies of isolates X1, X26, and X33, genes that are responsible for metal transport and metal-binding proteins, which play a role in the metal adaptation in microorganisms, were searched from the genomes of the three strains and compared with each other. The genome sequences of isolates X1, X26, and X33 were deposited at GenBank under the accession numbers CP132290, JAUZEF000000000, and CP132291, respectively.

## References

[1] Hacioglu N, Tosunoglu M. 2014. Determination of antimicrobial and heavy metal resistance profiles of some bacteria isolated from aquatic amphibian and reptile species. Environ Monit Assess 186:407–413. doi:10.1007/s10661-013-3385-y 23959346

[2] Cidre I, Pulido RP, Burgos MJG, Gálvez A, Lucas R. 2017. Copper and zinc tolerance in bacteria isolated from fresh produce. J Food Prot 80:969–975. doi:10.4315/0362-028X.JFP-16-513 28467185

[3] Shylla L, Barik SK, Joshi SR. 2021. Characterization and bioremediation potential of native heavy-metal tolerant bacteria isolated from rat-hole coal mine environment. Arch Microbiol 203:2379–2392. doi:10.1007/s00203-021-02218-5 33665708

[4] Squadrone S. 2020. Water environments: metal-tolerant and antibiotic-resistant bacteria. Environ Monit Assess 192:238. doi:10.1007/s10661-020-8191-8 32173770

[5] Jing Y, He Z, Yang X. 2007. Role of soil rhizobacteria in phytoremediation of heavy metal contaminated soils. J Zhejiang Univ Sci B 8:192–207. doi:10.1631/jzus.2007.B0192 17323432 PMC1810380

[6] Afridi MS, Van Hamme J d., Bundschuh J, Khan MN, Salam A, Waqar M, Munis MFH, Chaudhary HJ, Sumaira. 2021. Biotechnological approaches in agriculture and environmental management - bacterium Kocuria rhizophila 14ASP as heavy metal and salt- tolerant plant growth- promoting strain. Biologia 76:3091–3105. doi:10.1007/s11756-021-00826-6

[7] Oziegbe O, Oluduro AO, Oziegbe EJ, Ahuekwe EF, Olorunsola SJ. 2021. Assessment of heavy metal bioremediation potential of bacterial isolates from landfill soils. Saudi J Biol Sci 28:3948–3956. doi:10.1016/j.sjbs.2021.03.072 34220251 PMC8241888

[8] Díaz A, Marrero J, Cabrera G, Coto O, Gómez JM. 2022. Biosorption of nickel, cobalt, zinc and copper ions by Serratia marcescens strain 16 in mono and multimetallic systems. Biodegradation 33:33–43. doi:10.1007/s10532-021-09964-9 34657229 PMC8803796

[9] Lu M, Jiao S, Gao E, Song X, Li Z, Hao X, Rensing C, Wei G. 2017. Transcriptome response to heavy metals in Sinorhizobium meliloti CCNWSX0020 reveals new metal resistance determinants that also promote bioremediation by Medicago lupulina in metal-contaminated soil. Appl Environ Microbiol 83:e01244-17. doi:10.1128/AEM.01244-17 28778889 PMC5626999

[10] Khan AR, Park GS, Asaf S, Hong SJ, Jung BK, Shin JH. 2017. Complete genome analysis of Serratia marcescens RSC-14: a plant growth-promoting bacterium that alleviates cadmium stress in host plants. PLoS One 12:e0171534. doi:10.1371/journal.pone.0171534 28187139 PMC5302809

[11] Abbaszade G, Szabó A, Vajna B, Farkas R, Szabó C, Tóth E. 2020. Whole genome sequence analysis of Cupriavidus campinensis S14E4C, a heavy metal resistant bacterium. Mol Biol Rep 47:3973–3985. doi:10.1007/s11033-020-05490-8 32406019 PMC7239810

[12] Halema AA, El-Beltagi HS, Al-Dossary O, Alsubaie B, Henawy AR, Rezk AA, Almutairi HH, Mohamed AA, Elarabi NI, Abdelhadi AA. 2024. Omics technology draws a comprehensive heavy metal resistance strategy in bacteria. World J Microbiol Biotechnol 40:193. doi:10.1007/s11274-024-04005-y 38709343

[13] Srivastava P, Kowshik M. 2013. Mechanisms of metal resistance and homeostasis in haloarchaea. Archaea 2013:732864. doi:10.1155/2013/732864 23533331 PMC3600143

[14] Sullivan MJ, Terán I, Goh KGK, Ulett GC. 2024. Resisting death by metal: metabolism and Cu/Zn homeostasis in bacteria. Emerg Top Life Sci 8:45–56. doi:10.1042/ETLS20230115 38362914 PMC10903455

[15] Perry RD, Bobrov AG, Fetherston JD. 2015. The role of transition metal transporters for iron, zinc, manganese, and copper in the pathogenesis of Yersinia pestis. Metallomics 7:965–978. doi:10.1039/c4mt00332b 25891079 PMC4464991

[16] Akbari MS, Doran KS, Burcham LR. 2022. Metal homeostasis in pathogenic streptococci. Microorganisms 10:1501. doi:10.3390/microorganisms10081501 35893559 PMC9331361

[17] Chin N, Frey J, Chang CF, Chang YF. 1996. Identification of a locus involved in the utilization of iron by Actinobacillus pleuropneumoniae. FEMS Microbiol Lett 143:1–6. doi:10.1111/j.1574-6968.1996.tb08452.x 8807793

[18] Yousuf S, Karlinsey JE, Neville SL, McDevitt CA, Libby SJ, Fang FC, Frawley ER. 2020. Manganese import protects Salmonella enterica serovar Typhimurium against nitrosative stress. Metallomics 12:1791–1801. doi:10.1039/d0mt00178c 33078811 PMC7677218

[19] Peng ED, Lyman LR, Schmitt MP. 2021. Analysis of the manganese and MntR regulon in Corynebacterium diphtheriae. J Bacteriol 203:e0027421. doi:10.1128/JB.00274-21 34370555 PMC8459757

[20] Giachino A, Waldron KJ. 2020. Copper tolerance in bacteria requires the activation of multiple accessory pathways. Mol Microbiol 114:377–390. doi:10.1111/mmi.14522 32329112

[21] Groisman EA, Duprey A, Choi J. 2021. How the PhoP/PhoQ system controls virulence and Mg^2+^ homeostasis: lessons in signal transduction, pathogenesis, physiology, and evolution. Microbiol Mol Biol Rev 85:e0017620. doi:10.1128/MMBR.00176-20 34191587 PMC8483708

[22] Ali MK, Li X, Tang Q, Liu X, Chen F, Xiao J, Ali M, Chou S-H, He J. 2017. Regulation of inducible potassium transporter KdpFABC by the KdpD/KdpE two-component system in Mycobacterium smegmatis. Front Microbiol 8:570. doi:10.3389/fmicb.2017.00570 28484428 PMC5401905

[23] Chen D-D, Tian Y, Jiao J-Y, Zhang X-T, Zhang Y-G, Dong Z-Y, Xiong M-J, Xiao M, Shu W-S, Li W-J. 2020. Comparative genomics analysis of Nitriliruptoria reveals the genomic differences and salt adaptation strategies. Extremophiles 24:249–264. doi:10.1007/s00792-019-01150-3 31820112

[24] Hiramatsu T, Kodama K, Kuroda T, Mizushima T, Tsuchiya T. 1998. A putative multisubunit Na^+^/H^+^ antiporter from Staphylococcus aureus. J Bacteriol 180:6642–6648. doi:10.1128/JB.180.24.6642-6648.1998 9852009 PMC107768

[25] Ohyama T, Igarashi K, Kobayashi H. 1994. Physiological role of the chaA gene in sodium and calcium circulations at a high pH in Escherichia coli. J Bacteriol 176:4311–4315. doi:10.1128/jb.176.14.4311-4315.1994 8021217 PMC205643

[26] Thompson CC, Chimetto L, Edwards RA, Swings J, Stackebrandt E, Thompson FL. 2013. Microbial genomic taxonomy. BMC Genomics 14:913. doi:10.1186/1471-2164-14-913 24365132 PMC3879651

[27] Barboza NR, Morais MMCA, Queiroz PS, Amorim SS, Guerra-Sá R, Leão VA. 2017. High manganese tolerance and biooxidation ability of Serratia marcescens isolated from manganese mine water in Minas Gerais, Brazil. Front Microbiol 8:1946. doi:10.3389/fmicb.2017.01946 29062307 PMC5640716

[28] Ka-ot AL, Banerjee S, Haldar G, Joshi SR. 2018. Acid and heavy metal tolerant Bacillus sp. from rat-hole coal mines of Meghalaya, India. Proc Natl Acad Sci India Sect B Biol Sci 88:1187–1198. doi:10.1007/s40011-017-0856-x

[29] Elkenawy NM, Youssef NH, Aziz RK, Amin MA, Yassin AS. 2021. Draft genome sequence of a prodigiosin-hyperproducing Serratia marcescens strain isolated from Cairo, Egypt. G3 (Bethesda) 11:11. doi:10.1093/g3journal/jkab284 PMC8473970 34568929

[30] Van der Veken D, Hollanders C, Verce M, Michiels C, Ballet S, Weckx S, Leroy F. 2022. Genome-based characterization of a plasmid-associated micrococcin P1 biosynthetic gene cluster and virulence factors in Mammaliicoccus sciuri IMDO-S72. Appl Environ Microbiol 88:e0208821. doi:10.1128/AEM.02088-21 34936836 PMC8863057

[31] Wernimont AK, Huffman DL, Finney LA, Demeler B, O’Halloran TV, Rosenzweig AC. 2003. Crystal structure and dimerization equilibria of PcoC, a methionine-rich copper resistance protein from Escherichia coli. J Biol Inorg Chem 8:185–194. doi:10.1007/s00775-002-0404-9 12459914

[32] Achard MES, Tree JJ, Holden JA, Simpfendorfer KR, Wijburg OLC, Strugnell RA, Schembri MA, Sweet MJ, Jennings MP, McEwan AG. 2010. The multi-copper-ion oxidase CueO of Salmonella enterica serovar Typhimurium is required for systemic virulence. Infect Immun 78:2312–2319. doi:10.1128/IAI.01208-09 20231415 PMC2863522

[33] Gupta SD, Lee BT, Camakaris J, Wu HC. 1995. Identification of cutC and cutF (nlpE) genes involved in copper tolerance in Escherichia coli. J Bacteriol 177:4207–4215. doi:10.1128/jb.177.15.4207-4215.1995 7635807 PMC177164

[34] Staehlin BM, Gibbons JG, Rokas A, O’Halloran TV, Slot JC. 2016. Evolution of a heavy metal homeostasis/resistance island reflects increasing copper stress in Enterobacteria. Genome Biol Evol 8:811–826. doi:10.1093/gbe/evw031 26893455 PMC4824010

[35] Cabrera G, Xiong A, Uebel M, Singh VK, Jayaswal RK. 2001. Molecular characterization of the iron-hydroxamate uptake system in Staphylococcus aureus. Appl Environ Microbiol 67:1001–1003. doi:10.1128/AEM.67.2.1001-1003.2001 11157278 PMC92682

[36] Roy EM, Griffith KL. 2017. Characterization of a novel iron acquisition activity that coordinates the iron response with population density under iron-replete conditions in Bacillus subtilis. J Bacteriol 199:e00487-16. doi:10.1128/JB.00487-16 27795321 PMC5165090

[37] Gómez-Garzón C, Barrick JE, Payne SM. 2022. Disentangling the evolutionary history of Feo, the major ferrous iron transport system in bacteria. mBio 13:e0351221. doi:10.1128/mbio.03512-21 35012344 PMC8749426

[38] Janakiraman A, Slauch JM. 2000. The putative iron transport system SitABCD encoded on SPI1 is required for full virulence of Salmonella typhimurium. Mol Microbiol 35:1146–1155. doi:10.1046/j.1365-2958.2000.01783.x 10712695

[39] Schwartz CJ, Giel JL, Patschkowski T, Luther C, Ruzicka FJ, Beinert H, Kiley PJ. 2001. IscR, an Fe-S cluster-containing transcription factor, represses expression of Escherichia coli genes encoding Fe-S cluster assembly proteins. Proc Natl Acad Sci U S A 98:14895–14900. doi:10.1073/pnas.251550898 11742080 PMC64955

[40] Py B, Gerez C, Huguenot A, Vidaud C, Fontecave M, Ollagnier de Choudens S, Barras F. 2018. The ErpA/NfuA complex builds an oxidation-resistant Fe-S cluster delivery pathway. J Biol Chem 293:7689–7702. doi:10.1074/jbc.RA118.002160 29626095 PMC5961054

[41] Schwarz MGA, Antunes D, Corrêa PR, da Silva-Gonçalves AJ, Malaga W, Caffarena ER, Guilhot C, Mendonça-Lima L. 2020. Mycobacterium tuberculosis and M. bovis BCG moreau fumarate reductase operons produce different polypeptides that may be related to non-canonical functions. Front Microbiol 11:624121. doi:10.3389/fmicb.2020.624121 33510737 PMC7835394

[42] Winter MG, Hughes ER, Muramatsu MK, Jimenez AG, Chanin RB, Spiga L, Gillis CC, McClelland M, Andrews-Polymenis H, Winter SE. 2023. Formate oxidation in the intestinal mucus layer enhances fitness of Salmonella enterica serovar Typhimurium. mBio 14:e0092123. doi:10.1128/mbio.00921-23 37498116 PMC10470504

[43] Cellier MFM. 2022. Nramp: deprive and conquer? Front Cell Dev Biol 10:988866. doi:10.3389/fcell.2022.988866 36313567 PMC9606685

[44] Sharma G, Merz KM. 2021. Formation of the metal-binding core of the ZRT/IRT-like protein (ZIP) family zinc transporter. Biochemistry 60:2727–2738. doi:10.1021/acs.biochem.1c00415 34455776 PMC9002132

[45] Reynolds PE, Arias CA, Courvalin P. 1999. Gene vanXYC encodes D,D -dipeptidase (VanX) and D,D-carboxypeptidase (VanY) activities in vancomycin-resistant Enterococcus gallinarum BM4174. Mol Microbiol 34:341–349. doi:10.1046/j.1365-2958.1999.01604.x 10564477

[46] Jeudy S, Claverie JM, Abergel C. 2004. Crystal structure of a zinc-type alcohol dehydrogenase-like protein YahK. PDB. doi:10.2210/pdb1UUF/pdb

[47] Tomita A, Zhang M, Jin F, Zhuang W, Takeda H, Maruyama T, Osawa M, Hashimoto K, Kawasaki H, Ito K, Dohmae N, Ishitani R, Shimada I, Yan Z, Hattori M, Nureki O. 2017. ATP-dependent modulation of MgtE in Mg^2+^ homeostasis. Nat Commun 8:148. doi:10.1038/s41467-017-00082-w 28747715 PMC5529423

[48] Papp-Wallace KM, Maguire ME. 2008. Magnesium transport and magnesium homeostasis. EcoSal Plus 3. doi:10.1128/ecosalplus.5.4.4.2 26443723

[49] Matsushita T, Hirata H, Kusaka I. 1988. Calcium channel blockers inhibit bacterial chemotaxis. FEBS Lett 236:437–440. doi:10.1016/0014-5793(88)80072-3 3137094

[50] Rhoads DB, Waters FB, Epstein W. 1976. Cation transport in Escherichia coli. VIII. Potassium transport mutants. J Gen Physiol 67:325–341. doi:10.1085/jgp.67.3.325 4578 PMC2214971

[51] Mesbah NM, Wiegel J. 2011. The Na^+^-translocating F_1_F_O_-ATPase from the halophilic, alkalithermophile Natranaerobius thermophilus. Biochim Biophys Acta 1807:1133–1142. doi:10.1016/j.bbabio.2011.05.001 21600188

[52] Juárez O, Barquera B. 2012. Insights into the mechanism of electron transfer and sodium translocation of the Na^+^-pumping NADH:quinone oxidoreductase. Biochim Biophys Acta 1817:1823–1832. doi:10.1016/j.bbabio.2012.03.017 22465856 PMC8172247

[53] Kuhns M, Trifunović D, Huber H, Müller V. 2020. The Rnf complex is a Na^+^ coupled respiratory enzyme in a fermenting bacterium, Thermotoga maritima. Commun Biol 3:431. doi:10.1038/s42003-020-01158-y 32770029 PMC7414866

[54] Tamura K, Dudley J, Nei M, Kumar S. 2007. MEGA4: molecular evolutionary genetics analysis (MEGA) software version 4.0. Mol Biol Evol 24:1596–1599. doi:10.1093/molbev/msm092 17488738

[55] Koren S, Walenz BP, Berlin K, Miller JR, Bergman NH, Phillippy AM. 2017. Canu: scalable and accurate long-read assembly via adaptive k-mer weighting and repeat separation. Genome Res 27:722–736. doi:10.1101/gr.215087.116 28298431 PMC5411767

[56] Chen Z, Erickson DL, Meng JH. 2020. Benchmarking long-read assemblers for genomic analyses of bacterial pathogens using oxford nanopore sequencing. Int J Mol Sci 21:9161. doi:10.3390/ijms21239161 33271875 PMC7730629

[57] Hunt M, Silva ND, Otto TD, Parkhill J, Keane JA, Harris SR. 2015. Circlator: automated circularization of genome assemblies using long sequencing reads. Genome Biol 16:294. doi:10.1186/s13059-015-0849-0 26714481 PMC4699355

[58] Hyatt D, Chen G-L, Locascio PF, Land ML, Larimer FW, Hauser LJ. 2010. Prodigal: prokaryotic gene recognition and translation initiation site identification. BMC Bioinformatics 11:119. doi:10.1186/1471-2105-11-119 20211023 PMC2848648

[59] Tarailo-Graovac M, Chen NS. 2009. Using RepeatMasker to identify repetitive elements in genomic sequences. Curr Protoc Bioinformatics Chapter 4:4. doi:10.1002/0471250953.bi0410s25 19274634

[60] Chan PP, Lowe TM. 2019. tRNAscan-SE: searching for tRNA genes in genomic sequences. Methods Mol Biol 1962:1–14. doi:10.1007/978-1-4939-9173-0_1 31020551 PMC6768409

[61] Nawrocki EP, Eddy SR. 2013. Infernal 1.1: 100-fold faster RNA homology searches. Bioinformatics 29:2933–2935. doi:10.1093/bioinformatics/btt509 24008419 PMC3810854

[62] Rangannan V, Bansal M. 2009. Relative stability of DNA as a generic criterion for promoter prediction: whole genome annotation of microbial genomes with varying nucleotide base composition. Mol Biosyst 5:1758–1769. doi:10.1039/B906535K 19593472

[63] Blin K, Shaw S, Steinke K, Villebro R, Ziemert N, Lee SY, Medema MH, Weber T. 2019. antiSMASH 5.0: updates to the secondary metabolite genome mining pipeline. Nucleic Acids Res 47:W81–W87. doi:10.1093/nar/gkz310 31032519 PMC6602434

[64] Aziz RK, Bartels D, Best AA, DeJongh M, Disz T, Edwards RA, Formsma K, Gerdes S, Glass EM, Kubal M, et al.. 2008. The RAST Server: rapid annotations using subsystems technology. BMC Genomics 9:75. doi:10.1186/1471-2164-9-75 18261238 PMC2265698

